# Validation of Lower Urinary Tract Symptom Questionnaire in the Transmasculine Population

**DOI:** 10.1007/s00192-024-05895-0

**Published:** 2024-08-26

**Authors:** Fabiana M. Kreines, Caroline K. Cox, Sunni L. Mumford, Heidi S. Harvie, Lily A. Arya, Uduak U. Andy

**Affiliations:** 1 Division of Urogynecology, Department of Obstetrics and Gynecology, University of Pennsylvania, Philadelphia, PA USA; 2 Division of Urogynecology, Department of Obstetrics and Gynecology, University of Wisconsin, Madison, WI USA; 3 Department of Biostatistics, Epidemiology and Informatics, University of Pennsylvania, Philadelphia, PA USA

**Keywords:** LGBTQ, Transgender, Urogynecology

## Abstract

**Introduction and Hypothesis:**

Transgender men and transmasculine individuals report a variety of lower urinary tract symptoms (LUTS), but little is known about LUTS in this population. One of the obstacles is the lack of validated questionnaires. This study was aimed at validating the International Consultation on Incontinence Questionnaire–Lower Urinary Tract Symptoms (ICIQ-LUTS), which measures filling, voiding, and incontinence symptoms, in transmasculine individuals.

**Methods:**

This is an observational validation study that included transmasculine individuals receiving care within a single tertiary care hospital system. Construct validity was assessed by comparing the ICIQ-LUTS with severity of LUTS as measured by the Urinary Distress Inventory–Short Form (UDI-6), and concurrent validity by the association between ICIQ-LUTS and the Patient Perception of Bladder Condition (PPBC). Discriminant validity was determined by comparing ICIQ-LUTS scores in those with and those without self-reported LUTS. Spearman correlation, *t* test, and Kruskal–Wallis test were used for data analysis.

**Results:**

A total of 131 respondents were included in the analysis. Only two individuals (1.5%) reported prior vaginectomy and/or phalloplasty. Concurrent validity was demonstrated by a significant association between ICIQ-LUTS subscales and PPBC (filling *p* < 0.001, voiding *p* < 0.001, incontinence *p* < 0.001). Construct validity was demonstrated by a significant correlation between ICIQ-LUTS and UDI-6 (filling ρ = 0.76, *p* < 0.001; voiding ρ = 0.48, *p* < 0.001; incontinence ρ = 0.61, *p* < 0.001). For discriminant validity, those with at least one self-reported LUTS had significantly higher (worse) ICIQ-LUTS subscale scores than those without self-reported LUTS.

**Conclusions:**

The ICIQ-LUTS is valid for measurement of LUTS severity in transmasculine individuals. This will be an important tool to use in future research to learn more about LUTS in this population.

## Introduction

Transgender and gender diverse individuals are those whose gender identity differs from their assigned sex at birth. In the USA alone, it is estimated that 1.6 million people aged 13 years and older, 0.6% of the population, identify as transgender [1]. The number of people who self-identify as transgender and gender diverse has increased in recent years. Many transgender individuals seek out experts in the medical community for gender-affirming care, which can range from knowledgeable and affirming preventative care to hormone therapy and surgery.

Lower urinary tract symptoms (LUTS) are among the reasons why this population may seek medical care. Although transgender men and transmasculine (TGM/TM) individuals assigned female at birth report LUTS [2, 3], such as frequency, urgency, nocturia, and incontinence, there is little to no research investigating the rate or cause of these symptoms. For both transmasculine and transfeminine individuals, most research regarding lower urinary tract function centers around postoperative symptoms after gender-affirming surgery [4–8]. In transmasculine individuals specifically, LUTS such as urinary frequency, nocturia, and urinary incontinence have often been reported after genital gender-affirming surgery in as many as two thirds of individuals in some studies [4, 5]. Unfortunately, one of the obstacles in studying LUTS in this population is the lack of validated questionnaires. There are very few validated questionnaires for use in the transgender population in general, and those that do exist focus on mental health, body image, and resilience [9–13]. Without a validated questionnaire for TGM/TM individuals, all data regarding rates of LUTS and the effect of hormones and other such interventions on LUTS come from their cisgender counterparts.

Several instruments have been developed and validated to measure LUTS in cisgender women. The Sixth International Consultation on Incontinence (ICI) recommends usage of questionnaires from the International Consultation on Incontinence Questionnaire (ICIQ) modules for the evaluation of LUTS in clinical practice and for research purposes [14]. The International Consultation on Incontinence Questionnaire–Female Lower Urinary Tract Symptoms (ICIQ-FLUTS) allows for the assessment of the severity of a variety of LUTS, has been translated into several languages, and found to be applicable in several diverse populations [15–21]. It has been validated in cisgender women for use in nonsurgical and surgical populations, has been shown to be easily completed, to have good reliability, and be responsive to change [22]. Additionally, although it was intended for use in cisgender females, there is no mention of anatomy or gender in the questions; as it is being validated in a transmasculine population, it will be referred to as the ICIQ-LUTS. Given the known reliability and validity of the ICIQ-LUTS instrument in the cisgender population [22], the aim of this study was to evaluate the validity of the ICIQ-LUTS to diagnose and measure LUTS in the transmasculine population.

## Materials and Methods

### Study Design

This is an observational validation study that included TGM/TM individuals receiving care with OB/GYN and Family Practice within a single tertiary care hospital system. Our objective was to validate the ICIQ-LUTS in the TGM/TM population.

This study was reviewed and considered to be exempt by the University of Pennsylvania School of Medicine Institutional Review Board.

### Variables and Data Collection

Inclusion criteria were individuals assigned female at birth (including transmen, transmasculine individuals, nonbinary or other gender-diverse individuals), age 18 and older, and patients of at least one of four Attending Physicians in either Obstetrics and Gynecology or Family Medicine within a single tertiary care hospital system. To be included, individuals had to have received care with one of these providers between 1 January 2018 and 1 May 2023. Exclusion criteria were an inactive patient portal in the electronic health record (EHR) and an inability to read or speak English. All invitations and survey responses were conducted electronically. After electronic written consent was obtained, individuals were provided with a link to an online survey to provide sociodemographic information (including age, sex assigned at birth, gender, race, ethnicity, obstetric history, testosterone use history) and complete a series of questionnaires administered via the Research Electronic Data Capture (REDCap) system. Additional demographic information and medical comorbidities were collected from the patient’s electronic medical records. Participants were asked to complete the following questionnaires: the ICIQ-LUTS, Urinary Distress Inventory Short Form (UDI-6), and the Patient Perception of Bladder Condition (PPBC).

### Validation Process

The ICIQ-LUTS consists of 12 questions regarding the severity of LUTS on three separate subscales, scores of which range from 0 to 16 for symptoms of filling, 0 to 12 for voiding symptoms, and 0 to 20 for incontinence symptoms. This questionnaire measures the prevalence, frequency, and degree of bother of each individual LUTS [22]. Higher score indicates more severe symptoms.

As there is currently no gold standard to measure LUTS in the transgender population, validity was measured by comparing ICIQ-LUTS with instruments that are widely used and validated in both cisgender male and cisgender female populations. Construct validity, the ability of an instrument to measure the construct under investigation, was assessed by comparing the ICIQ-LUTS score with bother from LUTS as measured by the Urinary Distress Inventory–Short Form (UDI-6) using Spearman correlation. The UDI-6 is a six-item questionnaire that assesses the presence of and distress caused by LUTS. It is scored with a range from 0 to 75. It has been validated in both cisgender women and cisgender men [23, 24]. A higher score indicates a greater disability. If valid, we expected higher (worse) UDI-6 scores to correlate with higher (worse) ICIQ-LUTS subscale scores.

Concurrent validity, the relationship of an instrument to other similar instruments, was assessed by measuring the association between the ICIQ-LUTS and the PPBC. The PPBC is a single-item questionnaire that measures a patient’s subjective perception of their LUTS. Scores range from 1 to 6 (higher score indicating more severe symptoms). It has been validated in both cisgender women and cisgender men [25]. We compared PPCC scores with ICIQ-LUTS subscale scores using the Kruskal–Wallis test. If valid, we expected individuals with higher (more severe) PPBC scores to have higher (worse) mean ICIQ-LUTS subscale scores.

Discriminant validity, the ability of an instrument to distinguish between distinct populations, was determined by comparing ICIQ-LUTS scores in those with and those without self-reported LUTS. Self-reported LUTS were gathered from individual responses to each question on the UDI-6, which asks specifically about the presence of urinary frequency, urgency incontinence, stress incontinence, small amounts of leakage, difficulty emptying the bladder, and pain/discomfort in the lower abdominal/genital area. Those who reported that they were bothered “moderately” or more by any single LUTS were considered to have LUTS. Comparisons were made using a *t* test (two-sided). If valid, we expected individuals with at least one LUTS that was moderately or more bothersome to have higher (worse) mean ICIQ-LUTS subscale scores than those who did not.

### Statistics

Demographic data are presented as percentages or means ± standard deviation. Observed significant correlations < 0.3 were considered low, between 0.3 and 0.5 were considered moderate, and ≥ 0.5 were considered high, based on previously established cutoff points [26]. For validation of an instrument, a > 5:1 subject-to-item ratio is recommended [27]. As the ICIQ-LUTS questionnaire contains 12 questions, at least 60 participants were needed to provide sufficient power to validate this instrument. Spearman correlation, *t* test, and Kruskal–Wallis test were used for data analysis. Internal consistency was measured with Cronbach’s alpha. All statistics were performed in STATA version 18.0 (StataCorp LP, College Station, TX, USA). Statistical significance was defined as *p* < 0.05 for all parameters.

## Results

In all, 395 individuals were invited to participate. Two were subsequently excluded because they failed to meet the inclusion criteria owing to errors in information in the electronic medical record (one individual identified as a cisgender woman and one as a transgender woman assigned male at birth). Of the 393 individuals invited to participate who met the inclusion criteria, 131 elected to participate (response rate 33.3%). When demographics from the electronic medical records were compared in those who elected to participate and those who did not, there was no difference in age, BMI, race, ethnicity, prior hysterectomy, prior vaginectomy and/or phalloplasty, proportion who had previously been pregnant, proportion who had previously delivered a pregnancy, or proportion with a vaginal estrogen prescription. Those who elected to participate were significantly more likely to have a testosterone prescription (*p* = 0.001).

### Participant Demographics

One hundred and thirty-one participants were included in the analysis. Demographics are shown in Table 1. One hundred and six participants (80.9%) were taking testosterone for gender affirmation at the time of study participation. Of those taking testosterone, 31 (29.3%) had stopped and restarted testosterone at some point in the past. Of those not taking testosterone at the time of study participation, 12 (48.0%) had taken testosterone in the past. Thirty-six participants (27.5%) had had a prior hysterectomy, 26 (19.9%) had had a prior bilateral oophorectomy, 2 (1.5%) and had had a vaginectomy and/or phalloplasty. Only 19 (14.5%) participants had been pregnant in the past. Of these, 9 (47.4%) had delivered a pregnancy, 8 of whom had delivered at least one pregnancy vaginally.

**Table 1:** Patient demographics and lower urinary tract symptom (LUTS) scores (*n* = 131)

Demographic	Data
Age, mean ± SD (range)	30.4 ± 7.2 (19–63)
BMI, mean ± SD	29.4 ± 7.1 kg/m^2^
Race, number (%)	
White	108 (82.4)
Black	12 (9.2)
Asian	2 (1.5)
Other	9 (6.9)
Ethnicity, number (%)	
Hispanic	15 (11.5)
Obstetric history, number (%)	
Parous	9 (6.9)
History of vaginal delivery, number (%)	8 (6.1)
Gender identity, number (%)*	
Man	69 (52.7)
Nonbinary	56 (42.8)
Genderqueer	34 (26.0)
Other	14 (10.7)
Woman	2 (1.5)
Surgical history, number (%)	
Hysterectomy	36 (27.5)
Bilateral oophorectomy	26 (19.9)
Vaginectomy and/or phalloplasty	2 (1.5)
Questionnaire, mean ± SD (range)	
ICIQ-Filling	3.1 ± 2.6 (0–12)
ICIQ-Voiding	2.5 ± 2.4 (0–10)
ICIQ-Incontinence	2.2 ± 3.0 (0–19)
UDI-6	18.0 ± 16.2 (0–75)
PPBC, number (%)	
1	55 (42.0)
2	35 (26.7)
3	25 (19.1)
4	10 (7.6)
5	6 (4.6)

Patient demographic and overall lower urinary tract symptoms as measured by different questionnaires. *Participants could choose more than one answer for gender identity

ICIQ-LUTS consists of 12 questions regarding the severity of LUTS on three separate subscales, scores of which range from 0 to 16 for symptoms of filling, 0 to 12 for voiding symptoms, and 0 to 20 for incontinence symptoms

PPBC grades: 1 = “My bladder condition does not cause me any problems at all,” 2 = “some very minor problems,” 3 = “some minor problems, 4 = “(some) moderate problems,” 5 = “severe problems,” 6 = “many severe problems” (the latter was not selected by any participant)

*ICIQ-LUTS* International Consultation on Incontinence Questionnaire–Lower Urinary Tract Symptoms, *UDI-6* Urinary Distress Inventory–Short Form, *PPBC* Patient Perception of Bladder Condition

Overall questionnaire scores are also included in Table 1. Mean ICIQ-LUTS scores for the filling, voiding, and incontinence subscale scores were 3.1 ± 2.5, 2.5 ± 2.4, and 2.2 ± 3.0 respectively. Internal consistency of the 12 ICIQ-LUTS questions, as measured by Cronbach’s alpha, was α = 0.80.

### Construct Validity

Construct validity was confirmed with a significant correlation between all three ICIQ-LUTS subscale scores and UDI-6, as shown in Table 2. For all three subscales, *p* < 0.001. Spearman’s rank correlation coefficient was as follows: ICIQ-Filling ρ = 0.76; ICIQ-Voiding ρ = 0.48; ICIQ-Incontinence ρ = 0.61.

**Table 2:** Construct Validity: International Consultation on Incontinence Questionnaire–Lower Urinary Tract Symptoms (ICIQ-LUTS) correlation with Urinary Distress Inventory–Short Form (UDI-6)

	UDI-6 score	
	ρ	*p* value
ICIQ-F	0.76	< 0.001
ICIQ-V	0.48	< 0.001
ICIQ-I	0.61	< 0.001

Construct validity: ICIQ-LUTS correlation with UDI-6

Subscales: *F* filling, *V* voiding, *I* incontinence

Statistical analysis performed using Spearman correlation

### Concurrent Validity

**Fig. 1:**
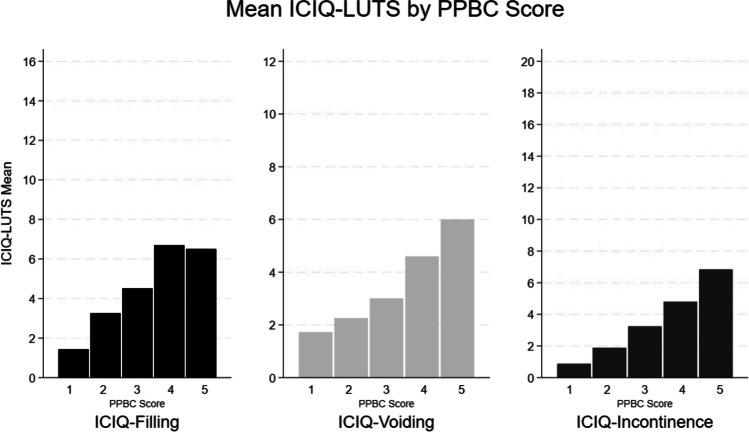
Concurrent validity: mean International Consultation on Incontinence Questionnaire–Lower Urinary Tract Symptoms (ICIQ-LUTS) subscale scores by Patient perception of Bladder Condition (PPBC) score. Analysis done using the Kruskal–Wallis test, *p* < 0.001 for all three subscales

Concurrent validity was demonstrated by significantly higher (worse) ICIQ-LUTS subscale scores in individuals reporting higher (more severe) PPBC scores (*p* < 0.001 with Kruskal–Wallis for all three ICIQ subscales), as shown in Fig. 1.

### Discriminant Validity

Overall, 67 (51.1%) self-reported to have at least one LUTS that was moderately bothersome or worse; 64 (48.9%) denied having any LUTS that were at least moderately bothersome. For assessment of discriminant validity, we noted that those with at least one self-reported LUTS had higher (worse) scores than those without self-reported LUTS for all three ICIQ-LUTS subscales, as shown in Table 3.

**Table 3:** Discriminant validity: mean International Consultation on Incontinence Questionnaire–Lower Urinary Tract Symptoms (ICIQ-LUTS) scores by presence or absence of self-reported lower urinary tract symptoms (LUTS)

	No or mild LUTS	Moderate/severe LUTS	
	Mean (± SD)	Mean (± SD)	*p* value
ICIQ-F	1.7 ± 1.6	4.5 ± 2.6	< 0.001
ICIQ-V	1.7 ± 1.7	3.3 ± 2.7	< 0.001
ICIQ-I	1.0 ± 1.6	3.3 ± 3.6	< 0.001

ICIQ-LUTS correlation with self-reported bladder symptoms. Those who reported that they were bothered “moderately” or more by any single LUTS on the UDI-6 were considered to have LUTS

Subscales: *F* filling, *V* voiding, *I* incontinence

*UDI-6* Urinary Distress Inventory–Short Form

## Discussion

Our findings demonstrate that the ICIQ-LUTS is a valid questionnaire for evaluation of LUTS severity in our TGM/TM population. We found that the ICIQ-LUTS scores correlated strongly with the UDI-6 score, confirming construct validity, as well as the PPBC score, confirming concurrent validity in our study population. We also found that participants with self-reported LUTS had worse ICIQ-LUTS scores than those without, confirming discriminant validity. These results support the use of the ICIQ-LUTS in the TGM/TM population to validly measure LUTS and extend research in this area.

To our knowledge, the only questionnaire regarding LUTS that had been validated prior to this study in the transgender population is the AFFIRM tool, which was created specifically for transfeminine individuals after gender-affirming surgery [4]. We believe that the lack of validated questionnaires has contributed to the dearth of research concerning LUTS in transgender individuals. We have now validated the ICIQ-LUTS in this transmasculine population, which will hopefully allow measurement of LUTS and support research in this population. Of note, as we conducted our validation in a population with a very small proportion who had previously had a vaginectomy and/or phalloplasty (1.5% of participants), these results can likely only be generalized to populations who have not undergone genital gender-affirming surgery.

In a recent qualitative study of transfeminine individuals, several participants reported LUTS such as urge incontinence, stress incontinence, retention, and recurrent urinary tract infections [2]. The study population had variable prior gender-affirming treatments (medical and surgical); however, the reports of LUTS were captured qualitatively and the severity of symptoms was not assessed. Our validation of the ICIQ-LUTS will now allow quantitative assessment of LUTS severity in this population. Further, future analyses can likely be done to validate its use in capturing change in symptom severity following a variety of medical and surgical treatments in this population.

A strength of this study is the relatively large sample size used to validate the ICIQ-LUTS. Additionally, our validation of the ICIQ-LUTS is strengthened by the fact that discriminant, construct, and concurrent validity were all confirmed. This study also has several limitations. First, as previously mentioned, only two participants had a history of vaginectomy and/or phalloplasty, which limits generalizability to a postoperative population. Second, the overall rates and scores of LUTS in this population were low (only 31% of patients reported bladder problems that were a minor problem or worse on the PPBC), which may limit the generalizability of these findings to a population with more severe and bothersome LUTS. Third, although we measured the presence and severity of many different types of LUTS in our evaluation of discriminant validity (such as urinary frequency, urgency incontinence, stress incontinence, small amounts of leakage, difficulty emptying the bladder, and pain/discomfort in the lower abdominal/genital area), it is important to note that this list is not exhaustive and did not include items such as urinary tract infection, nocturia, etc. It is therefore possible that some important LUTS relevant to the transmasculine population were not captured in this instrument. Fourth, the response rate to our invitation for study participation was low at 33%. A concern with a low response rate is that the population surveyed is not representative of the population at large owing to response bias. However, when we compared individuals who chose to participate in our study with those who did not, we did not find any significant difference in demographics between the two groups. Those who did participate, though, were significantly more likely to have a prescription for testosterone. This discrepancy may be because the invitation to participate in the study included the word “testosterone” in the title, as additional studies from this cohort will examine the effects of testosterone. Unfortunately, this may have selectively encouraged those currently using testosterone to participate. The relationship between gender-affirming testosterone use and LUTS is currently not known, so it is difficult to ascertain how this may affect our results. Future analysis will explore this association further. Finally, traditional validation studies are usually done by comparing the results of the instrument against a gold standard; however, no such gold standard exists for the evaluation of LUTS in the transgender population. Nonetheless, we chose the UDI-6 and PPBC as these instruments are widely used and validated in both cisgender male and cisgender female populations.

Future studies will need to further evaluate the ICIQ-LUTS in this population, including obtaining patient input to assess that it sufficiently addresses the range of LUTS experienced by transgender individuals and adequately captures their specific needs. It will also be important to calculate the minimal important difference and sensitivity for use in this population, as well as confirm the validity of this instrument in the TGM/TM population after genital gender-affirming surgery. Future validation efforts should also be extended to the transfeminine population.

In conclusion, the ICIQ-LUTS is a valid questionnaire for evaluation of LUTS severity in the pre-operative TGM/TM population and can be used in the future to measure LUTS in this population for both clinical and research purposes. This will be important to accurately evaluate LUTS in the TGM/TM population, which, until now, has not had a validated method of measuring LUTS clinically.

## Data Availability

The data that support the findings of this study are available from the corresponding author, F.M.K., upon reasonable request.
